# Selection and Interpretation of Scientific Evidence in Preparation for Policy Decisions: A Case Study Regarding Introduction of Rotavirus Vaccine Into National Immunization Programs in Sweden, Norway, Finland, and Denmark

**DOI:** 10.3389/fpubh.2018.00131

**Published:** 2018-05-14

**Authors:** Gry St-Martin, Ann Lindstrand, Synne Sandbu, Thea Kølsen Fischer

**Affiliations:** ^1^Virology Surveillance and Research Unit, Statens Serum Institut, Copenhagen, Denmark; ^2^The Public Health Agency of Sweden, Stockholm, Sweden; ^3^Department of Vaccine Preventable Diseases, Norwegian Institute of Public Health, Oslo, Norway; ^4^Center for Global Health, Department of Infectious Diseases, University of Southern Denmark, Odense, Denmark

**Keywords:** policy decisions, vaccine introduction, rotavirus vaccines, Nordic countries, policy-making processes

## Abstract

The World Health Organization recommends inclusion of rotavirus vaccines in national immunization programs (NIPs) worldwide. Nordic countries are usually considered comparable in terms of demographics and health-care services and have comparable rotavirus disease burden. Nevertheless, the countries have reached different decisions regarding rotavirus vaccine: Norway and Finland have already introduced rotavirus vaccines into their NIPs and Sweden is currently changing its recommendation and vaccines will now be introduced on a national scale while Denmark has decided against it. This study focuses on the selection and interpretation of medical and epidemiological evidence used during the decision-making processes in Sweden, Norway, Finland, and Denmark. The so-called “severity criteria” is identified as one of the main reasons for the different policy decisions reached across the Nordic countries.

## Introduction

Two oral, live, attenuated rotavirus vaccines, Rotarix™ (derived from a human G1P[8] rotavirus strain) and RotaTeq™ (a reassorted bovine–human rotavirus), are used internationally; and both vaccines are considered highly effective in preventing severe gastrointestinal disease (1). The World Health Organization recommends that all countries include rotavirus vaccines in their national immunization program (NIP) (2), and as of February 2017, 87 countries have done so, including 16 in the European Region (3).

Globally, there are no uniform guidelines for decision-making processes or policies for introduction of new vaccines, although individual countries usually consider disease burden, health care and vaccine-related costs and safety and effectiveness of vaccine candidates (4). Surveillance and research studies on rotavirus hospitalizations and deaths (5) have demonstrated that the incidence of rotavirus does not vary much across countries worldwide, but the risk of dying from rotavirus disease is much higher in low-income countries, and there is general agreement on the rationale for the need for prevention of rotavirus disease in such settings (6). In high-income countries, where rotavirus-associated mortality is generally low, there has been more debate on the need for and cost-effectiveness (CE) of the vaccines (7).

The Nordic countries are usually considered similar and comparable in terms of demographics, disease burden and health-care systems with tax-based funding, publicly owned and operated hospitals, universal residence-based access, and comprehensive coverage (8).

In addition, their setups for evidence assessment are similar, in that all countries establish working groups formalized and chaired by national public health institutions and with broad representation from medical and public health communities. These working groups formulate their interpretation of the evidence as well as recommendations for the policy makers. The final decision to introduce the vaccine is then taken by the national government. Nevertheless, the countries have reached different decisions in terms of introducing rotavirus vaccines. Norway and Finland have introduced rotavirus vaccines into their NIP (9, 10), while Denmark has decided against it (11). At present, there is no national recommendation about rotavirus vaccine in Sweden but the Government has decided to introduce the rotavirus vaccine during 2019. However, Sweden’s 21 counties (so-called län: sub-national regions) have the option to provide vaccines free of charge in addition to the NIP, and to date, 8 of them have introduced the rotavirus vaccine without awaiting a national decision (12).

Public health policy decisions often involve assessment of scientific evidence. While policy processes are complicated and influenced by factors beyond scientific evidence (13), it is conceivable that differences in the use of evidence to inform policy may be a contributing factor to differences in policy between countries. The aim of the present study was to examine this issue in the context of introduction of rotavirus vaccine into the NIP in Sweden, Norway, Finland, and Denmark. The aim of the study was to identify if differences in the evidence base, or in the interpretation of it, contributed to differences in the policy decisions.

## Materials and Methods

The study was initiated in Denmark, and to obtain information on the decision processes in three other Nordic countries, we contacted middle- and/or top-level leaders at the national public health institutions who contributed the evidence base for the policy processes in Denmark, Sweden, Norway, and Finland, respectively. The email contained a brief introduction to the study along with a semi-structured questionnaire intended to facilitate the presentation of the evidence considerations. Further the main email included an invitation to forward the questionnaire (Table 1) to the person in the institution with most insight into the issue.

**Table 1:** Content of questionnaire addressing rotavirus vaccine decision-taking process in Norway, Sweden, Finland, and Denmark.

Category	Topics	Specified items
(1) Rotavirus epidemiology and disease burden	Annual rotavirus-associated hospitalizations and deaths Is rotavirus notifiable Is a surveillance system capturing rotavirus disease in place (national/regional level)	

(2) General vaccine related	Procedure for introducing new vaccines into the national program	Constellation of a national vaccine advising committee and/or relevant national authorities

(3) Rotavirus vaccine specific	When and how did rotavirus vaccines enter the health policy agenda in the country Which evidence was sought to support the policy process Was new evidence produced (e.g., disease burden study, economic evaluation, HTA) prior to the decision Which policy options were under consideration and the expected outcomes	(a) to introduce rotavirus vaccine in NIP free of charge, (b) to introduce the vaccine with payment/co-payment by recipients, (c) not to introduce in the general program but recommend/encourage parents to get their children vaccinated, (d) not to introduce in the general program and not recommend its use

(4) Society and acceptance	The public understanding and acceptance of the policy-making process and of the final decision	

*The questionnaire consisted mainly of open-ended questions (without ranking), divided into four categories addressing the following elements: (1) National estimates of rotavirus disease burden (specifically hospitalizations and deaths) and surveillance system in place for rotavirus infections, (2) general vaccine-related questions, including the procedure for introducing new vaccines into the national program including the potential constellation of a national vaccine advising committee and/or relevant national authorities, and (3) rotavirus vaccine-specific questions about when and how rotavirus vaccines have entered the health policy agenda in the country, which evidence was sought to support the policy process, if new evidence was produced [e.g., disease burden study, economic evaluation, health technology assessment (HTA)] prior to the decision, and which policy options were under consideration and the expected outcomes: (a) to introduce rotavirus vaccine in national immunization program (NIP) free of charge, (b) to introduce the vaccine with payment/co-payment by recipients, (c) not to introduce in the general program but recommend/encourage parents to get their children vaccinated, (d) not to introduce in the general program, and not recommend its use. Which criteria were employed to decide on the desirability of each outcome, how economic analyses have been incorporated, whether a health sector or a broader perspective was used, description of ethical concerns addressed in the policy process, brief discussion of main issues raised and main discussion points, and (4) how the public understanding and acceptance of the policy-making process and of the decision taken has been*.

The responses from each country were compared with regards to similarities and differences in the production and use of evidence to support the decisions taken. All discrepant views are presented in the result section to reduce any bias due to interpretations of the responses.

As a supplement to the information on vaccination policy provided in the questionnaire, a background literature on rotavirus vaccine policy in the four countries was retrieved from a PubMed search (using the keyword “rotavirus” combined with each of the four country names) and from the websites of national public health agencies of the countries.

## Results

### Sweden

Sweden will introduce universal rotavirus vaccine into the NIP in 2019. Rotavirus vaccines have been available for purchase at private vaccination centers for several years prior to this decision, and one of the vaccines available on the market is subsidized by the Dental and Pharmaceutical Benefits Agency, TLV. Furthermore, the County councils in Sweden have the possibility to offer vaccination free of charge to the population of that county, even if the vaccine is not part of the NIP, and as of February 2018, eight of them have decided to do so.

Decisions regarding inclusion of new vaccines in the Swedish NIP are taken by the Swedish government (14). To support the government’s decision, the Public Health Agency (Folkhälsomyndigheten) is required to provide the government with a proposition prior to any changes in the NIP. Swedish law on infectious disease control outlines the criteria for considerations of new vaccine introduction, including availability of a vaccine offering long-term protection in the target population and suitable for use without prior diagnostic tests, effective in preventing spread of the infection in the population, cost-effective from a societal perspective, and the vaccination program must be justifiable on ethical and humanitarian grounds. The Public Health Agency has established a reference group for the NIP composed of representatives from counties and municipal governments, professional associations, organizations charged with implementing vaccination programs as well as relevant authorities. For questions on specific vaccines, the public health agency establishes working groups composed of experts from relevant fields. The reference group comments on the assessments of the working group before the public health agency decides on the proposition to the government. In their proposition to the government, the Public Health Agency considers the burden and severity of disease, the likely impact of vaccination on disease burden, safety, dosing schedule, and feasibility of integration into the existing NIP, target groups, acceptability of the vaccine and the need for information and communication initiatives, the impact of vaccination on health-care sector and society including issues of social inequalities and access, and the possibilities for monitoring and surveillance (14).

Studies had demonstrated that disease burden and costs were largely comparable to other European countries and very few rotavirus-associated deaths were registered. National rotavirus disease burden estimates were assessed to 1–3 deaths among children less than 5 years over a 5-year period or <0.1 per 100,000 child-years (15, 16). An estimated 2,000 children, or 3.6 per 1,000 children, less than 5 years were hospitalized due to rotavirus infection before any county started rotavirus vaccination (17).

In 2015, The Public Health Agency published a report on disease burden (17), expected impact of vaccination and a plan for rotavirus infection surveillance as well as a health economic analysis of universal rotavirus vaccine (18) in preparation for the elaboration of an assessment and proposition to the government, which was finalized in 2017 (19). The health economic analysis showed that, from a societal perspective, where indirect costs were included in the analysis, the vaccine would be directly cost saving. From a health sector perspective, however, vaccines would be cost-effective but not directly cost saving (Table 2). The Public Health Agency has concluded that rotavirus vaccine meets the criteria set out in Swedish law for introducing the new vaccine based on the large number of cases among children less than 3 years old, including both hospitalized children and children managed at home with or without contact to outpatient services. In addition, the risk–benefit ratio was deemed favorable as the risk of intussusception is estimated to be low in Sweden and the condition is treatable in hospitals. The Public Health Agency also emphasized the findings from the health economic evaluation, the generally high acceptance of the existing NIP which has a coverage of 97–98% among 2-year olds. Finally, the Public Health Agency had sought the advice of the Swedish National Council on Medical Ethics which concluded that universal rotavirus vaccination would be ethically justifiable, provided caregivers receive adequate and well-balanced information and focus remains on the health and interest of the children and not the interests of parents, workplaces, and health budget. In February 2017, the Public Health Agency of Sweden concluded that vaccination against rotavirus infection fulfilled the requirements of the Communicable Diseases Act and should be included in the national vaccination program for children, and in December 2017, the government decided to introduce the vaccine in the NIP during 2019.

**Table 2:** Organization and outcomes of national decision processes on rotavirus vaccine introduction in Scandinavian countries (Denmark, Finland, Norway, and Sweden).

	Rotavirus vaccine included in the national childhood vaccination program	NITAG established	National decision process on vaccine introduction				
			Formal framework exists	Has cost-effectiveness (CE) analysis been applied	Results CE analysis, Societal perspective	Conclusion CE assessment health sector perspective	Main drivers for/against introduction
Denmark		+		+	Cost-effective when indirect costs included	Not cost-effective	Severity (mortality) criteria

Finland	+	+	+	+	“Reasonably” cost-effective	Not cost-effective	High morbidity burden, safe vaccines

Norway	+	+	+	+	Cost-effective when indirect costs included	Unlikely cost-effective	High morbidity burden

Sweden		+	+	+	Cost-effective and cost-saving when indirect costs included	Cost-effective but not cost-saving	High morbidity burden

*NITAG, National Immunization Technical Advisory Groups*.

### Norway

In Norway, the decision to introduce a new vaccine into the NIP is taken by the Ministry of Health and Care Services, based on advice from the NIPH (National Institute of Public Health). Considerations of the rotavirus vaccine for introduction into the NIP were initiated already in 2006 when the first vaccine candidates were licensed in Europe. Evaluation of the vaccine was done by a working group chaired by the NIPH and including representatives from the NIPH as well as from the scientific community and health professionals (doctors and nurses). Among the evidence considered by the group were studies on disease burden (20, 21).

Prior to vaccine introduction, an estimated 900 children, or 3.6 per 1,000 children less than 5 years, were hospitalized each year with rotavirus gastroenteritis in Norway and the mortality, although not specifically registered, was estimated as two deaths in children under 5 years old over a 5-year period. This shows that rotavirus disease in Norway hardly meets the severity criteria for a disease to be prevented by vaccination.

The NIPH included data from other high-income countries that had already introduced the vaccine and experienced important reductions in rotavirus incidence. Data from Mexico and Brazil pertaining to the risk of intussusception were cited to justify a favorable risk–benefit ratio. The NIPH found that the overall burden of disease, particularly the number of hospitalizations and outpatient visits, the safety of the vaccine, and the general acceptance and high coverage of the existing NIP were arguments to support the introduction of the vaccine. The working group included considerations about economic/social inequalities and health, such as whether children needing it most might not get a given vaccine if it is not free of charge. The conclusion of the majority of the working group was that the vaccine should be included in the NIP although one working group member, the representative of the national nurses’ association, assessed that the evidence for health as well as economic benefits from vaccination was insufficient.

A full economic analysis was done by a separate working group (22). The vaccine was found unlikely to be cost-effective in Norway from a health sector perspective. Further, the economic savings related to cases prevented would occur in hospitals and general practitioners’ offices, while the added costs of implementing the program would be incurred by the municipality health stations where vaccines would be administered. When indirect costs were included, the vaccine would likely be cost-effective (22). Indirect costs comprised loss of productivity related to parents’ absence from work. In addition, in the final recommendation (9), the NIPH estimated that the vaccine price would be lower after introduction than the price on which the cost–benefit analysis was based, and hence the vaccine was expected to become more cost-effective. Part of the recommendation was to include establishment of rotavirus surveillance, costs related to dissemination of information to health professionals and general population in the budget.

The National Council for Priority Setting in Health Care (which existed until the end of 2017) discussed the issue in June 2012 and decided, with a narrow majority, that though the vaccine was safe and effective and not very expensive, the disease was not considered serious enough to justify vaccination of all infants in Norway. However, in 2013, based on the existing evidence the Ministry of Health and Care Services took a political decision to introduce rotavirus vaccine in the NIP, with implementation from autumn 2014.

### Denmark

In Denmark, the decision to introduce new vaccines into the NIP is taken by the Ministry of Health based on advice from the national vaccine advisory committee, which is a group of experts led by the Danish Health Authority with representatives from clinical, laboratory, and epidemiological services and health authorities. The criteria considered when deciding on the possible introduction of a new vaccine include disease burden, severity of the disease targeted by the vaccine, safety and effectiveness of the vaccine.

In 2011, following the publication of two independent studies showing a considerable burden of rotavirus disease requiring hospitalization as well as requests from vaccine producers and clinical societies, the national vaccine advisory committee requested the Danish Health Authority to conduct a health technology assessment (HTA). The HTA included scientific evidence, health economic analyses, and qualitative research (focus group interviews with parents) and was published in 2012 (23). The economic evaluations concluded that introduction of vaccines would only be cost-effective from a societal perspective when including the indirect costs (Table 2). Even though rotavirus disease was documented to lead to ~1,200 hospitalizations annually among approximately 325,000 children <5 years of age and an incidence of 3.8 hospitalizations per 1,000 children <5 years of age annually (24), the vast majority of hospitalized children with no underlying disease were expected to recover from the disease without severe complications. Rotavirus was assessed to cause one death every 5 year in Denmark based on extrapolation of data from the USA (23).

According to the Danish Health Authority, the purpose of Denmark’s childhood vaccination program is to protect children from diseases that can result in either death or long-term harm, using safe and effective vaccines. It is stated explicitly that Denmark should not implement vaccinations in the NIP merely because this is feasible but only when the disease has serious consequences for individuals and/or society—the so-called “severity criterion.” Rotavirus infection hardly ever results in death or long-term harm in countries such as Denmark with free and universal access to health care.

The vaccine advisory committee and the Danish Health Authority decided not to recommend the introduction rotavirus vaccine in the NIP due to failure to fulfill the severity criterion (11).

### Finland

Finland was invited to participate in this study, but only epidemiological data were returned in reply to the questionnaire, and we sought additional information through publicly available information and articles from scientific journals.

Finland was the first Nordic country and one of the first countries in Europe to include rotavirus vaccine in the NIP in 2009 (10). In Finland, a national advisory committee for vaccination under the National Public Health Institute which establishes an expert group when new vaccines are considered for introduction. The expert group will base its evaluation on four criteria established by the national advisory committee. These criteria must be met before introduction of a new vaccine into the NIP in Finland and include: burden of disease and the potential of the vaccine for reducing the burden; safety of the vaccine for individuals; no adverse effects of the vaccine at the population level; and CE from a societal point of view (25). A health economic evaluation was carried out, showing the vaccine to be “not cost-saving but reasonably cost-effective, especially if nosocomial infections and home-treated rotavirus cases were included” (10) (Table 2). Some of the research leading to the approval of the two rotavirus vaccines was carried out in Finland, and the studies demonstrated a high safety and effectiveness of the vaccine within the Finnish population (25).

## Policy Options and Implications

In summary, this study illustrates how four countries with comparable public health-care systems and similar burden of rotavirus disease arrive at different decisions regarding potential introduction of rotavirus vaccine in the NIP. Differences in use of the evidence to inform the decision-making process seem to have played a role for the different conclusions reached. Most importantly, there are different conceptions of whether the consequences of disease for the individual and/or society as a whole are substantial enough to justify universal vaccination, exposing healthy children to an intervention, and whether it merits the added expenses on the national health-care budget.

As far as this study shows, all countries include disease burden as a key criterion, but interpret it differently: Denmark considered the low mortality and benign course of most cases of the infection to be an argument against introduction, whereas Norway, Sweden, and Finland consider the number of cases and health-care visits to be an indicator of a considerable disease burden.

Another main difference between the countries is seen in their health economic evaluations. CE analyses are always sensitive to model assumptions, to decisions about discounting and about whether to include costs and benefits outside the health-care sector, and analyses concerning vaccines perhaps even more so for several reasons. The possible adverse effects of vaccination usually occur shortly after vaccination, while the disease prevented by the vaccine might occur years ahead in the future, and hence benefits of vaccines are discounted more than its disadvantages. For childhood diseases, the models used in the economic evaluation in different countries may differ in regard to whether time spent by parents caring for the sick child, income lost to parents, and/or productivity losses in society are considered in the analyses. Herd immunity is also not consistently included in the analyses. Finally, quality of life measures typically included in economic evaluations have not been validated in children (26, 27). As noted by Bruggenjurgen (27): “Indeed, most studies have found RV vaccination to be cost-effective in certain scenarios only, for example when the effects of herd immunity and a societal perspective are considered” (p. 2290). The same article also notes that “while there is some evidence demonstrating a high burden to care givers, there is a need for further research data to more accurately quantify the economic impact of this, as this can have a considerable impact on the findings of cost-effectiveness analyses” (p. 2292). Economic evaluations from different European countries have reached different conclusions (28–30). Vaccine introduction is more likely to be deemed cost-effective when indirect costs and not only direct health care-associated costs are taken into consideration. Assumptions about the actual price of the vaccine in the context of a national vaccination program (i.e., how much the price will be reduced after national tender and negotiations) can also alter the conclusion about CE. Health care-associated costs also vary considerably between countries based on estimates of hospitalization costs, nosocomial transmission, and modeling of incidence. For the indirect costs, the conclusion of the analysis is dependent on various parameters such as parental absenteeism from work, parents’ expenses to babysitters, etc. Such differences in health economic analyses between countries are deemed to manifest themselves in different vaccination policies.

**Figure 1:**
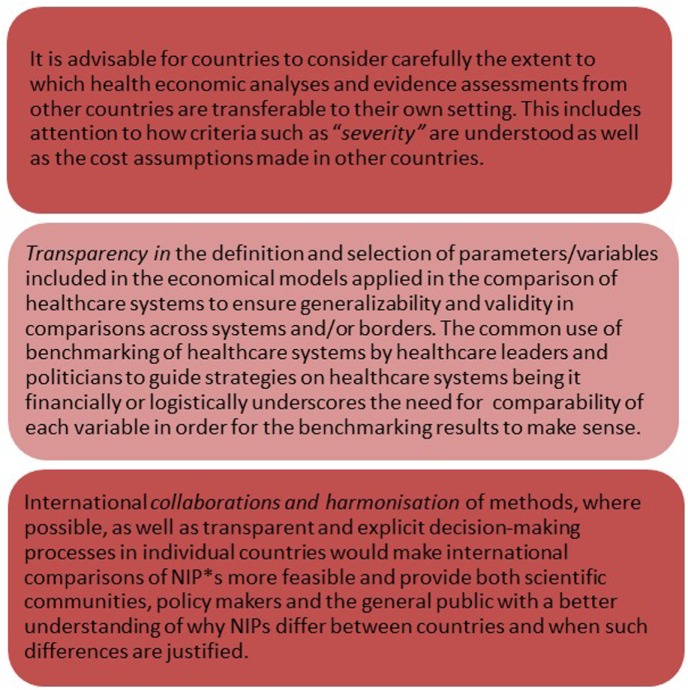
Actionable recommendations. Abbreviation: NIP, national immunization programs.

Although initiatives have been launched to harmonize economic evaluation methodology and make the process more comparable and transparent (31) (Figure 1), differences in national results could still arise from the fact that the costs and other actual values used for the calculations will to some extent be country specific as described above. Most European countries have established technical advisory groups (NITAGs) that provide advice and recommendations to decision makers regarding vaccine policies and programs, and many countries have well described frameworks for decision-making and include the same overall considerations in their vaccine policy decisions. Consequently, some scope exists for supranational harmonization although differences in NIPs will remain due to country specific priorities and data and variations in existing NIPs and health system structure (32, 33). In addition, different composition, analysis frameworks and work processes in countries’ NITAGs probably also contribute to differences in their recommendations to national decision makers (34).

## Conclusion

In conclusion, decisions on whether to introduce a vaccine into a country’s NIP are not based solely on available scientific evidence including health economic assessment [9]. As this study has shown, the scientific evidence itself is also not used and interpreted consistently across countries. Consequently, even comparable countries with very similar infrastructure, public health-care systems, disease burden, and cost-assessments arrive at different decisions when they interpret the same international, scientific evidence in the context of their national priorities and traditions (e.g., the “severity criterion”) and carry out economic evaluations using different models and specific national inputs (e.g., costs).

## Author Contributions

GS-M and TF conceptualized and designed the study and analyzed the data. GS-M wrote the first draft and TF revised all versions. AL and SS provided data and input to all versions of the manuscript.

## Conflict of Interest Statement

The authors declare that the research was conducted in the absence of any commercial or financial relationships that could be construed as a potential conflict of interest.
